# 

**Published:** 1903-12-19

**Authors:** 


CONTENTS.

Epilepsy and Crime - - - - - - - - - - 199

The Origin of Cancer - - - - - - - - - 200

Annotations - - - - - - - - - - - - 201

Notes and News - - - - - - - - - - - 202

Hospital Clinics and Medical Progress—

What We Owe to Experiments on Animals - - - - - - 203

Syphilitic Affections of the Heart and Aorta - - - - - 204

Idiosyncrasy in Relation to Doses - - - - - - - - 205

The Condition of the Pupil in Acute Alcoholic Poisoning - - 206

Progress in Cancer - - - - - - - - - - 206

Progress in Fevers - - - - - - - - - - 207

Progress in Neurology - - - - - - - - - 208

New Appliances and Things Medical - - - - 209

The Book World of Medicine and Science - - 210

Hospital Administration—

King Edward's Hospital Fund for London - - - - - - 211

The Metropolitan Hospital Sunday Fund - - - - - - 215

St. Bartholomew's Hospital - - - - - - - - 217

Glances at the Hospitals - - - - - - - - 217

The Student of Medicine - - - - - - - - 218

*NURSING SECTION.*

Notes on News from the Nursing World—
Our Christmas Distribution—Christmas Preparations at the London Hospitals—Princess Louise and Jubilee Nurses—Lady Dudley's Appeal—The Coronation Nursing Fund—The Resignations at Charing Cross Hospital—Glasgow and West of Scotland Co-operation of Trained Nurses—Nurses and Hospital Construction—Alleged Scandal at Hackney Workhouse Infirmary—An Injustice to Midwives trained in Ireland—Rescue of a Nurse by a Matron at Hull—Nurses and Nerve Training—American Indiscretion—A Trained Nurse for Granard Hospital—Dublin Nurses and their Physician—Examination at Bristol General Hospital—Examination at Kingston Infirmary—Short Items - 159-162
Lectures upon the Nursing of Mental Diseases 163
District Nursing and Diet - - - - - - - 164
The Nurses of Darlington Hospital - - - - 165
Appointments - - - - - - - - - - 166
Christmas Books - - - - - - - - - - 167
Novelties for Nurses - - - - - - - - 168
Presentations - - - - - - - - - - - 168
"The Hospital" Convalescent Fund - - - - 168
Everybody's Opinion - - - - - - - - 169
Miss Florence Nightingale and Belfast Nurses 170
Echoes from the Outside World - - - - - 171
Notes and Queries - - - - - - - - - 172
For Reading to the Sick - - - - - - - - 172

